# Meta-analysis of flavonoids use into beef and dairy cattle diet: Performance, antioxidant status, ruminal fermentation, meat quality, and milk composition

**DOI:** 10.3389/fvets.2023.1134925

**Published:** 2023-02-15

**Authors:** José Felipe Orzuna-Orzuna, Griselda Dorantes-Iturbide, Alejandro Lara-Bueno, Alfonso Juventino Chay-Canul, Luis Alberto Miranda-Romero, Germán David Mendoza-Martínez

**Affiliations:** ^1^Departamento de Zootecnia, Universidad Autónoma Chapingo, Texcoco, Mexico; ^2^División Académica de Ciencias Agropecuarias, Universidad Juárez Autónoma de Tabasco, Villahermosa, Mexico; ^3^Departamento de Producción Agrícola y Animal, Universidad Autónoma Metropolitana—Xochimilco, Mexico City, Mexico

**Keywords:** oxidative stress, natural antioxidants, natural phytochemicals, immunity, digestibility

## Abstract

The objective of this study was to evaluate the effects of dietary supplementation with flavonoids (FLAs) on animal performance, diet digestibility, antioxidant status in blood serum, rumen parameters, meat quality, and milk composition in beef and dairy cattle through a meta-analysis. Thirty-six peer-reviewed publications were included in the data set. The weighted mean differences (WMD) between the FLAs treatments and the control treatment were used to assess the effect size. Dietary supplementation with FLAs decreased feed conversion ratio (WMD = −0.340 kg/kg; *p* = 0.050) and increased (*p* < 0.05) dry matter intake (WMD = 0.191 kg/d), dry matter digestibility (WMD = 15.283 g/kg of DM), and daily weight gain (WMD = 0.061 kg/d). In blood serum, FLAs supplementation decreased the serum concentration of malondialdehyde (WMD = −0.779 nmol/mL; *p* < 0.001) and increased (*p* < 0.01) the serum concentration of superoxide dismutase (WMD = 8.516 U/mL), glutathione peroxidase (WMD = 12.400 U/mL) and total antioxidant capacity (WMD = 0.771 U/mL). A higher ruminal propionate concentration (WMD = 0.926 mol/100 mol; *p* = 008) was observed in response to FLAs supplementation. In meat, the dietary inclusion of FLAs decreased (*p* < 0.05) shear force (WMD = −1.018 kgf/cm^2^), malondialdehyde content (WMD = −0.080 mg/kg of meat), and yellowness (WMD = −0.460). Supplementation with FLAs decreased milk somatic cell count (WMD = −0.251 × 103 cells/mL; *p* < 0.001) and increased (*p* < 0.01) milk production (WMD = 1.348 kg/d), milk protein content (WMD = 0.080/100 g) and milk fat content (WMD = 0.142/100 g). In conclusion, dietary supplementation with FLAs improves animal performance and nutrient digestibility in cattle. In addition, FLAs improve the antioxidant status in blood serum and the quality of meat and milk.

## 1. Introduction

As part of the strategies to satisfy the growing demand for meat and dairy products, it is necessary to increase effectiveness and productivity in bovine production systems (1). Dairy cows and beef cattle are frequently exposed to a wide variety of stressors, such as environmental (heat or cold stress), physiological (for example, rapid growth rate), and nutritional (presence of mycotoxins or oxidized fat in diets) (2, 3). All these factors promote the overproduction of reactive oxygen species, alter the redox balance and cause oxidative stress in animals (4). Oxidative stress is associated with a higher incidence of diseases (5, 6) and leads to diminished cattle productive and reproductive performance (3). According to Abuelo et al. (7), dietary supplementation with exogenous antioxidants such as vitamins and trace elements can reduce oxidative stress and improve cattle's health status and productive performance. However, in recent years, interest in using natural antioxidants (not from chemical synthesis) as alternatives to the synthetic antioxidants commonly used in animal feed has increased (8). Potential natural antioxidants include flavonoids (FLAs). The FLAs consist of two benzene rings joined by three carbon atoms to form an oxygenated heterocycle (9) and are present in a wide variety of plants (10).

It has been documented that FLAs possess diverse biological properties, such as antioxidant, anti-inflammatory, hepatoprotective, and antimicrobial (10). The effects of dietary inclusion of FLAs have been investigated mainly in broilers and laying hens (11–13). However, in ruminants, there is limited information on the effects of dietary supplementation with FLAs. In growing ruminants, dietary supplementation with FLAs results in the reduction of diarrhea's occurrence and severity. However, it is ineffective in improving animal metabolism and productive performance (14). On the other hand, in adult ruminants, there is evidence that dietary supplementation with FLAs increases the serum concentration of antioxidant enzymes, reduces lipid peroxidation, and improves total antioxidant capacity in blood serum (15). In cattle and goats, some parts of plants containing FLAs have been used to increase the productive performance and digestibility of consumed nutrients (16, 17). Previous studies (18, 19) have shown that, in adult cattle, FLAs supplementation reduces agonistic interactions and modifies the differential expression of genes involved in inflammation, regulation of feeding behavior, and animal behavior. Specifically, in the ruminal epithelium of beef cattle, Paniagua et al. (19) detected greater gene expression of two genes (free fatty acid receptor 3 and free fatty acid receptor 2) that improve the feeding pattern in beef cattle by increasing the time the animals spend consuming forage and concentrate (18). Likewise, in adult sheep and cattle, it has been reported that the dietary inclusion of FLAs has a positive impact on the composition of the rumen microbiome and the production of volatile fatty acids in the rumen (20, 21).

Particularly in beef cattle and dairy cows, some studies have evaluated the effects of dietary supplementation with FLAs on animal performance (19, 22), serum antioxidant status (23, 24), rumen fermentation and nutrient digestibility (16, 20), meat physicochemical characteristics (25, 26) and milk production and composition (15, 27). However, the results obtained so far are still inconsistent and controversial, probably due to the wide variability among these studies regarding the experimental periods, the doses, and the type of FLAs used (14). Therefore, it is necessary to identify and control this variability to develop products containing FLAs that can improve the antioxidant status, animal performance, and quality of beef and dairy cattle products.

In recent years, some review articles have been published (9, 14), mentioning that it is possible to use FLAs for the improvement of the antioxidant status in blood serum, health status, animal performance, and quality of food products derived from ruminants. However, these review articles neither focus only on beef cattle or dairy cows nor used a meta-analytic approach. Meta-analysis (MA) is a method that allows previously published results of a series of individual studies to be collected, combined, and statistically analyzed (28). Likewise, the MA helps identify sources of heterogeneity between studies (29). Therefore, there is a growing interest in the application of MA in the field of animal nutrition (30). However, the use of MA in research related to the inclusion of natural feed additives in ruminant diets is still limited (31). The hypothesis of this meta-analysis states that adding FLAs in beef and dairy cattle diets will benefit animal performance, antioxidant status, and rumen parameters without affecting the quality of products derived from these animals. Therefore, the objective of this study was to evaluate the effects of dietary supplementation with flavonoids FLAs on animal performance, diet digestibility, serum antioxidant status, rumen parameters, meat quality, and milk composition derived from beef and dairy cattle through a meta-analysis.

## 2. Materials and methods

### 2.1. Literature search and study selection

**Figure 1:**
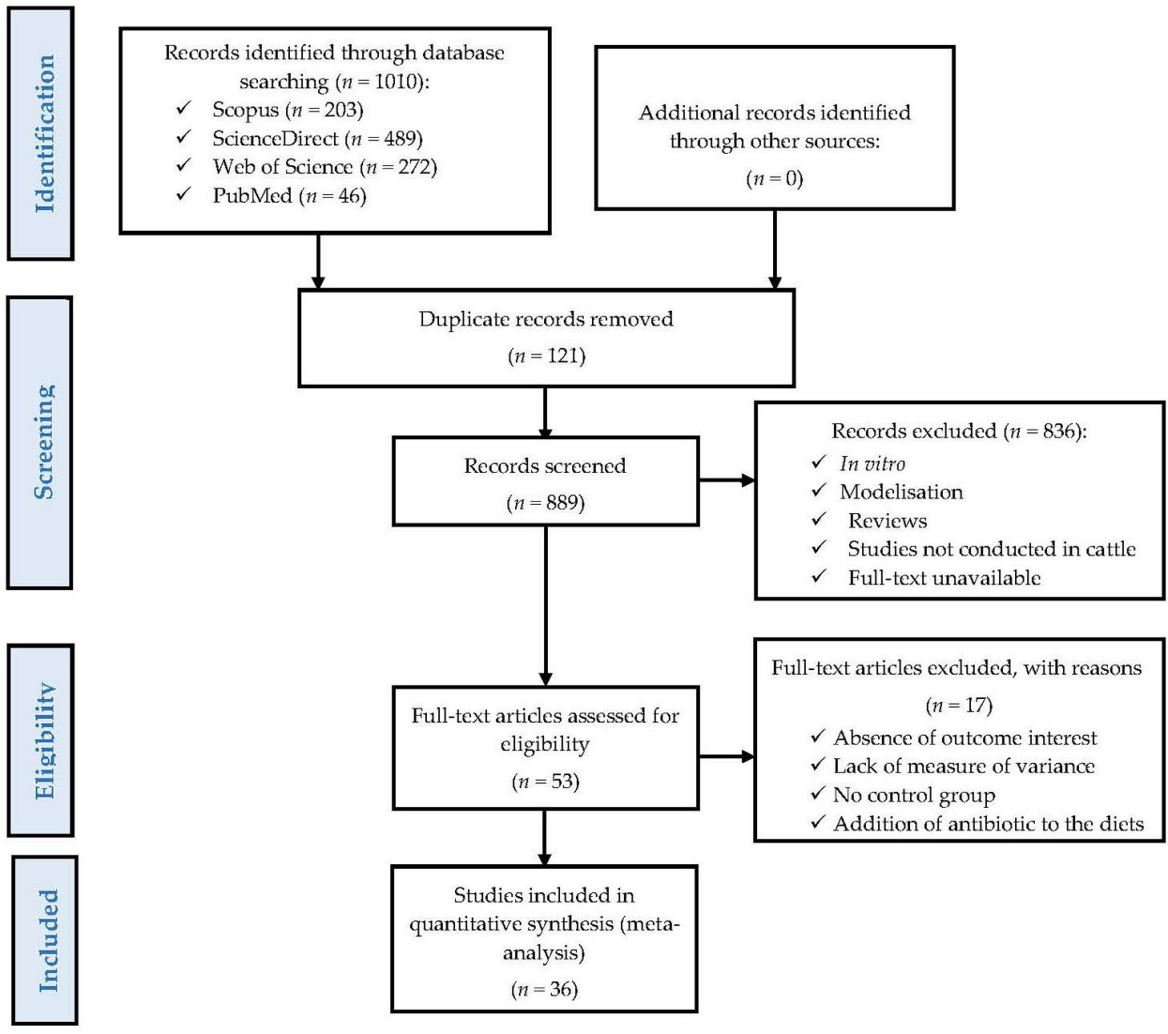
A PRISMA flow diagram detailing the literature search strategy and study selection for the meta-analysis.

To reduce publication bias and ensure the quality of the meta-analysis, the present study was conducted following PRISMA guidelines (32), as shown in Figure 1. A systematic search for information was conducted using Web of Science, Scopus, PubMed, and ScienceDirect databases to identify previous studies evaluating the effects of dietary supplementation with FLAs on nutrient digestibility, animal performance, carcass characteristics, antioxidant status in blood serum, ruminal fermentation, as well as meat and milk quality in beef (Holstein, Simmental, Angus × Nellore, Jinjiang, Xianan, and native) and dairy cattle (Holstein). The keywords that were used in all the databases were the following: “flavonoids, beef cattle, growth performance, finishing steer, finishing bull, carcass, meat quality, dairy cattle, milk production, milk quality, digestibility, ruminal fermentation, antioxidant status”, and the main representatives of FLAs (33), such as “daidzein, naringin, puerarin, anthocyanin, and quercetin”. In all searches performed, results were restricted to studies published between January 2010 and November 2022. In total, 1,010 scientific publications were identified (Figure 1); however, duplicate publications found in more than one of the databases were excluded. After this, the remaining publications underwent a two-step selection process, as previously reported by other authors (34–36). First, based on the titles and abstracts of each publication, we excluded studies that were not conducted in beef cattle or dairy cows, studies that did not measure any of the variables of interest, *in vitro* experiments, studies that used animals experimentally infected, as well as simulation and review articles.

Secondly, the articles analyzed had to meet some previously defined inclusion criteria to be included in the final database. In the present meta-analysis, the inclusion criteria used were similar to those previously reported by Dorantes-Iturbide et al. (35) and Orzuna-Orzuna et al. (36): (1) studies with beef cattle or dairy cows housed in confined conditions; (2) data on animal performance, nutrient digestibility, antioxidant status in blood serum, carcass characteristics, ruminal fermentation or quality of the derived products (meat or milk); (3) studies that had control and experimental treatments with similar diets, except for the presence of FLAs in the diets; (4) studies that reported the doses of FLAs used or had sufficient information to estimate the amount of FLAs included in the diets; (5) studies written and published in English and in peer-reviewed scientific journals; and (6) studies that reported the means of the control and FLA-supplemented treatments, the standard error or standard deviation, and the number of replicates.

### 2.2. Data extraction

After applying the inclusion criteria, only 36 peer-reviewed articles were included in the final database (Supplementary Table S1). Likewise, we only extracted quantitative data for response variables that were reported in at least three individual studies (31, 35, 36). Among the response variables included in the final database of this meta-analysis are the following: dry matter intake and nutrient digestibility (neutral detergent fiber, crude protein), daily weight gain, feed conversion ratio, carcass characteristics (carcass yield, backfat thickness), serum concentration of malondialdehyde and antioxidant enzymes (for example, superoxide dismutase), serum immunoglobulins (IgA, IgM, and IgG), rumen parameters (pH, ammonia nitrogen), physicochemical characteristics of the meat (pH, shear force, color), milk production, and milk composition (lactose, protein, and fat content).

Additionally, when available, the following complementary information was obtained from the selected publications: (1) author and year of publication; (2) period of supplementation with FLAs (days); (3) type of FLAs (for example, anthocyanin, daidzein); (4) method of inclusion of the FLAs (extract or naturally present in the diet); (5) amount of concentrate included in the diets (g/kg DM); (6) days in milk from dairy cows; (7) type of cattle (beef cattle or dairy cow); (8) nutritional composition of the diets used; and (9) country where the study was conducted.

Supplementary Table S1 shows the complete list of publications included in the final database of the present meta-analysis. The number of replicates, means, and standard deviations (SD) for the control and experimental treatments (supplemented with FLAs) were extracted from each of these publications. In all the publications in which the SD was not reported, SD was determined using the standard errors of the treatment means (SEM), by using the following equation (37): SD = SEM × √*n*, where *n* = number of repetitions.

### 2.3. Calculations and statistical analysis

Meta-analysis and meta-regression, as well as analyzes of subgroups, heterogeneity, and publication bias, were performed using the “metaphor” package (38), which is available in the statistical software R (version 4.1.2, R Core Team, Vienna, Austria). The effects of including FLAs in diets of beef cattle and dairy cows were evaluated using the weighted mean differences (WMD) between treatments supplemented with FLAs (diets with FLAs) and control treatments (diets without FLAs). For this, the means of the treatments were weighted by the inverse of the variance, according to the method for random effects models previously proposed by DerSimonian and Laird (39). In the present meta-analysis, the WMD was used because it allows interpretation of the results obtained in the original units of measurement (40). Additionally, with the PROC MEANS procedure of the statistical software SAS (41), descriptive statistics values were obtained for the continuous covariates level of concentrate in the diet, dose of FLAs, experimental period, and days in milk.

### 2.4. Heterogeneity and publication bias

The presence of heterogeneity between studies was identified with the chi-square (*Q*) test, in which a significance level of *p* ≤ 0.10 was used since this test has relatively low power (42). Additionally, to quantify the proportion of observed heterogeneity, we used the *I*^2^ statistic (29). For this test, *I*^2^ values <25% indicate that the degree of heterogeneity is low, *I*^2^ values between 25 and 50% indicate moderate heterogeneity, while *I*^2^ values >50% indicate high and significant heterogeneity (29, 43). On the other hand, to detect the presence of publication bias, the Egger regression asymmetry test (44) was applied, in which a significance level of *p* ≤ 0.05 was used. When publication bias was detected (*p* ≤ 0.05 in Egger's test), the “trim and fill” method of Duval and Tweedie (45) was applied to estimate the possible number of missing observations.

### 2.5. Meta-regression and subgroup analysis

Meta-regression analyses were performed on some of the variables evaluated to identify the presence of possible sources of heterogeneity. The criteria considered to apply meta-regression analysis were: (1) presence of significant heterogeneity (i.e., *p* ≤ 0.10 for Q or *I*^2^ > 50%); (2) *p*-value > 0.05 for Egger's test (44); and (3) response variables reported in 10 or more individual studies (46). For all meta-regression analyses, the method of moments of DerSimonian and Laird (39) was used, as it is well-established for estimating between-study variance. Subsequently, for the covariates analyzed that were significant with *p* ≤ 0.05, the WMD was evaluated through subgroup analysis. A subgroup assessment was not performed when an individual stratum has less than two effect sizes in the meta-analysis (35, 36). The type of FLAs (daidzein, anthocyanin, puerarin, naringin, quercetin, catechin, and blend), the method of supplementation with FLAs (extract or naturally present in an ingredient in the diet), and the type of cattle (beef cattle or dairy cow) were used as categorical covariates. On the other hand, the duration of the experimental period (days), the days in milk of the dairy cows, the content of concentrate in the diet (g/kg of DM), and the doses of FLAs were used as continuous covariates. When any categorical covariate (type of FLAs, type of bovine, and method of supplementation with FLAs) was found to be statistically significant (*p* ≤ 0.05), subgroup analysis was used to assess WMD (34, 35). Likewise, when the meta-regression was significant (*p* ≤ 0.05) for the continuous covariates, these were analyzed using the following subgroups: dietary dose of FLAs (≤600 and >600 mg/kg of DM), level of concentrate in the diet (≤400, 401–700, and >700 g/kg of DM), days in milk (≤100 and >100 days) and period of supplementation with FLAs (≤75 and >75 days).

## 3. Results

### 3.1. Study attributes

The studies included in this meta-analysis were conducted in eight different countries, mainly in China (44.4%), Spain (16.7%), Brazil (13.9%), and Japan (5.5%). Regarding the animal species (bovine), in 66.7% of the studies, beef cattle were used, and in the remaining studies (33.3%), dairy cows were used (Supplementary Table S1). Supplementary Table S2 shows that the doses of FLAs used were between 12 and 3,104 mg/kg DM. Dairy cows had between 7 and 164 days in milk and the experimental periods ranged between 24 and 168 days (Supplementary Table S2). Supplementary Table S1 shows seven different types of FLAs used in the present meta-analysis. Most of the studies used mixtures of FLAs (36.1%), daidzein (16.7%), anthocyanin (16.7%), and naringin (16.7%). Three other different types of FLAs were used in the remaining studies (13.8%). In addition, 61.1% of the studies used FLAs extracts, and 38.9% used plants or by-products naturally high in FLAs.

### 3.2. Dry matter intake and nutrient digestibility

Dry matter intake (DMI) increased in response to FLAs supplementation (Table 1). Likewise, dietary supplementation with FLAs increased (*p* < 0.05) dry matter digestibility (DMD), organic matter digestibility (OMD), crude protein digestibility (CPD), neutral detergent fiber digestibility (NDFD), acid detergent fiber digestibility (ADFD), and ether extract digestibility (EED).

**Table 1:** Dry matter intake and nutrient digestibility of cattle supplemented with flavonoids.

**Item**	***N* (NC)**				**Heterogeneity**		**Egger test^a^**
		**Control means (SD)**	**WMD (95% CI)**	***P*** **-value**	***P*** **-value**	*I*^2^ **(%)**	***P*** **-value**
DMI, kg/d	24 (43)	11.83 (5.08)	0.191 (0.047; 0.334)	0.009	<0.001	77.63	0.184
**Digestibility, g/kg of DM**							
DMD	11 (22)	687.84 (34.53)	15.283 (7.306; 23.259)	<0.001	0.348	0	0.112
OMD	10 (20)	712.10 (58.30)	7.204 (0.353; 14.055)	0.039	0.361	7.65	0.643
CPD	12 (25)	670.00 (90.10)	19.785 (13.099; 26.471)	<0.001	0.116	27.97	0.090
NDFD	12 (25)	523.30 (69.60)	15.563 (9.215; 21.911)	<0.001	0.723	0	0.377
ADFD	10 (20)	446.70 (104.80)	6.894 (0.524; 13.265)	0.034	0.417	3.19	0.883
EED	5 (8)	772.60 (87.80)	24.945 (8.962; 40.927)	0.002	0.498	0	NA

*N*, number of studies; NC, number of comparisons between flavonoids treatment and control treatment; SD, standard deviation; WMD, weighted mean differences between control and treatments with flavonoids; CI, confidence interval of WMD; p-value to χ2 (Q) test of heterogeneity; I^2^, proportion of total variation of size effect estimates that is due to heterogeneity.

a Egger's regression asymmetry test.

NA, variables with n < 10 observations, the test does not apply; DMI, dy matter intake; DMD, dry matter digestibility; OMI, organic matter digestibility; CPD, crude protein digestibility; EE, ether extract digestibility; NDFD, neutral detergent fiber digestibility; ADFD, acid detergent fiber digestibility; EED, ether extract digestibility.

### 3.3. Growth performance and carcass traits

Table 2 shows that daily weight gain (ADG) and backfat thickness (BFT) increased in response to dietary supplementation with FLAs (*p* < 0.05). In contrast, the dietary inclusion of FLAs decreased the feed conversion ratio (FCR; *p* = 0.050). However, hot carcass weight (HCW), hot carcass yield (HCY), and *Longissimus dorsi* muscle area (LDMA) were not affected by FLAs supplementation (*p* > 0.05; Table 2).

**Table 2:** Growth performance and carcass characteristics of cattle supplemented with flavonoids.

**Item**	***N* (NC)**				**Heterogeneity**		**Egger test^a^**
		**Control means (SD)**	**WMD (95% CI)**	***P*** **-value**	***P*** **-value**	*I*^2^ **(%)**	***P*** **-value**
ADG, kg/d	12 (22)	0.926 (0.41)	0.061 (0.026; 0.097)	<0.001	<0.001	73.47	0.657
FCR, kg/kg	9 (16)	7.90 (2.41)	−0.340 (−0.686; 0.005)	0.050	<0.001	81.77	0.122
**Carcass traits**							
HCW, kg	5 (5)	277.7 (68.8)	0.101 (−3.145; 3.347)	0.951	0.656	0	NA
HCY, %	6 (7)	54.56 (3.03)	−0.059 (−0.662; 0.544)	0.847	0.016	61.41	NA
BFT, mm	6 (8)	13.42 (3.63)	2.178 (0.829; 3.528)	0.002	<0.001	96.69	NA
LDMA, cm^2^	5 (7)	90.92 (19.98)	0.535 (−1.954; 3.025)	0.673	0.146	37.03	NA

*N*, number of studies; NC, number of comparisons between flavonoids treatment and control treatment; SD, standard deviation; WMD, weighted mean differences between control and treatments with flavonoids; CI, confidence interval of WMD; *p*-value to χ2 (*Q*) test of heterogeneity; *I*^2^, proportion of total variation of size effect estimates that is due to heterogeneity.

a Egger's regression asymmetry test.

NA, variables with *n* < 10 observations, the test does not apply; ADG, average daily gain; FCR, feed conversion ratio; HCW, hot carcass weight; HCY, hot carcass yield; BFT, backfat thickness; LDMA, *Longissimus dorsi* muscle area.

### 3.4. Antioxidant status and immune response

Table 3 shows that dietary supplementation with FLAs increased (*p* < 0.01) the serum concentration of superoxide dismutase (SOD), catalase (CAT), glutathione peroxidase (GPx), and total antioxidant capacity (TAC). In contrast, a lower (*p* < 0.001) serum concentration of malondialdehyde (MDA) was observed in animals supplemented with FLAs. On the other hand, the serum concentration of immunoglobulin A (IgA), immunoglobulin G (IgG), and immunoglobulin M (IgM) increased in response to dietary supplementation with FLAs (*p* < 0.01).

**Table 3:** Oxidative status and immune response of cattle supplemented with flavonoids.

**Item**	***N* (NC)**				**Heterogeneity**		**Egger test^a^**
		**Control means (SD)**	**RMD (95% CI)**	***P*** **-value**	***P*** **-value**	**I**^2^ **(%)**	***P*** **-value**
SOD, U/mL	10 (27)	72.39 (39.23)	8.516 (5.095; 11.937)	<0.001	<0.001	91.50	0.125
CAT, U/mL	3 (10)	39.26 (15.32)	3.762 (1.691; 5.833)	<0.001	<0.001	92.69	0.481
GPx, U/mL	7 (22)	63.73 (25.51)	12.400 (8.481; 16.319)	<0.001	<0.001	80.42	0.063
TAC, U/mL	5 (13)	7.86 (3.52)	0.771 (0.274; 1.267)	0.002	0.118	35.53	0.067
MDA, nmol/mL	7 (24)	5.51 (2.82)	−0.779 (−1.220; −0.339)	<0.001	<0.001	85.26	0.203
**Immunoglobulins, g/L**							
IgA	4 (14)	0.792 (0.14)	0.063 (0.018; 0.108)	0.006	0.001	61.32	0.492
IgG	5 (15)	9.137 (2.13)	1.150 (0.633; 1.667)	<0.001	<0.001	76.47	0.063
IgM	4 (14)	2.183 (0.59)	0.215 (0.139; 0.292)	<0.001	<0.001	73.86	0.400

*N*, number of studies; NC, number of comparisons between flavonoids treatment and control treatment; SD, standard deviation; WMD, weighted mean differences between control and treatments with flavonoids; CI, confidence interval of WMD; *p*-value to χ2 (*Q*) test of heterogeneity; *I*^2^, proportion of total variation of size effect estimates that is due to heterogeneity.

a Egger's regression asymmetry test.

SOD, superoxide dismutase; CAT, catalase; GPx, glutathione peroxidase; TAC, total antioxidant capacity; MDA, malondialdehyde; IgA, immunoglobulin A; IgG, immunoglobulin G; IgM, immunoglobulin M.

### 3.5. Rumen fermentation and protozoal count

Table 4 shows that the pH and the ruminal concentration of ammonia nitrogen (NH3-N), acetate, and butyrate were not affected by dietary supplementation with FLAs (*p* > 0.05). However, a higher (*p* = 0.008) rumen concentration of propionate and a lower (*p* = 0.023) concentration of total protozoa were observed in response to supplementation with FLAs.

**Table 4:** Ruminal fermentation of cattle supplemented with flavonoids.

**Item**	***N* (NC)**				**Heterogeneity**		**Egger test^a^**
		**Control means (SD)**	**RMD (95% CI)**	***P*** **-value**	***P*** **-value**	*I*^2^ **(%)**	***P*** **-value**
Ruminal pH	11 (20)	6.43 (0.46)	0.029 (−0.059; 0.117)	0.517	<0.001	83.29	0.129
NH_3_-N, mg/dL	9 (18)	15.03 (6.23)	0.030 (−0.559; 0.618)	0.921	<0.001	85.95	0.061
**SCFA, mol/100 mol**							
Acetate	12 (22)	62.12 (8.22)	0.188 (−0.794; 1.170)	0.708	<0.001	79.63	0.104
Propionate	12 (22)	22.34 (4.51)	0.926 (0.240; 1.611)	0.008	<0.001	89.01	0.261
Butyrate	12 (22)	11.72 (3.62)	0.138 (−0.105; 0.381)	0.265	0.237	17.09	0.086
Total protozoa, × 10^5^/mL	3 (8)	6.50 (2.40)	−0.301 (−0.561; −0.042)	0.023	0.054	49.37	NA

*N*, number of studies; NC, number of comparisons between flavonoids treatment and control treatment; SD, standard deviation; WMD, weighted mean differences between control and treatments with flavonoids; CI, confidence interval of WMD; *p*-value to χ2 (*Q*) test of heterogeneity; *I*^2^, proportion of total variation of size effect estimates that is due to heterogeneity.

a Egger's regression asymmetry test.

NA, variables with *n* < 10 observations, the test does not apply; NH_3_-N, nitrogen ammonia; SCFA, short chain fatty acids.

### 3.6. Meat quality

Dietary supplementation with FLAs did not affect (*p* > 0.05) pH, cooking loss (CL), lightness (L^*^), redness (a^*^), or meat protein, moisture, and ash content (Table 5). On the other hand, FLAs supplementation decreased (*p* < 0.05) the shear force (ShF), the malondialdehyde content (MDA), and the yellowness (b^*^) of the meat. However, meat's intramuscular fat content (IMF) increased in response to FLAs supplementation (*p* = 0.029).

**Table 5:** Meat quality of cattle supplemented with flavonoids.

**Item**	***N* (NC)**				**Heterogeneity**		**Egger test^a^**
		**Control means (SD)**	**WMD (95% CI)**	***P*** **-value**	***P*** **-value**	*I*^2^ **(%)**	***P*** **-value**
pH 24 h	6 (10)	5.47 (0.21)	0.063 (−0.032; 0.158)	0.194	<0.001	86.34	0.769
CL, g/100 g	4 (8)	23.54 (4.98)	0.628 (−1.433; 2.690)	0.550	0.028	55.52	NA
ShF, kgf/cm^2^	5 (8)	5.98 (2.54)	−1.018 (−1.470; −0.566)	<0.001	<0.001	88.17	NA
MDA, mg/kg	4 (8)	0.44 (0.16)	−0.080 (−0.101; −0.059)	<0.001	0.608	0	NA
**Meat color**							
Lightness (L^*^)	6 (13)	44.52 (10.32)	−2.174 (−5.117; 0.769)	0.148	<0.001	93.47	0.337
Redness (a^*^)	6 (13)	24.05 (6.62)	−0.065 (−0.734; 0.605)	0.850	0.177	26.42	0.669
Yellowness (b^*^)	6 (13)	10.65 (2.33)	−0.460 (−0.892; −0.028)	0.037	0.272	17.50	0.681
**Chemical composition, g/100 g of DM**							
Protein	6 (10)	20.61 (1.90)	0.390 (−0.622; 1.401)	0.450	<0.001	87.37	0.897
IMF	6 (10)	5.90 (2.52)	0.703 (0.070; 1.336)	0.029	<0.001	69.02	0.079
Moisture	6 (10)	70.28 (1.80)	−0.601 (−1.304; 0.101)	0.093	0.073	42.76	0.734
Ash	3 (5)	2.63 (1.17)	0.013 (−0.012; 0.039)	0.304	0.905	0	NA

*N*, number of studies; NC, number of comparisons between flavonoids treatment and control treatment; SD, standard deviation; WMD, weighted mean differences between control and treatments with flavonoids; CI, confidence interval of WMD; *p*-value to χ2 (*Q*) test of heterogeneity; *I*^2^, proportion of total variation of size effect estimates that is due to heterogeneity.

a Egger's regression asymmetry test.

NA, variables with *n* < 10 observations, the test does not apply; WHC, water holding capacity; CL, cook loss; ShF, shear force; MDA, malondialdehyde; IMF, intramuscular fat.

### 3.7. Milk production and composition

Dietary supplementation with FLAs increased (*p* < 0.01) milk production and milk protein and fat content (Table 6). However, the lactose content in milk was not affected by FLAs supplementation (*p* > 0.05). In addition, lower milk somatic cell (SCC) counts were observed in response to dietary supplementation with FLAs (*p* < 0.001).

**Table 6:** Milk production and quality of cattle supplemented with flavonoids.

**Item**	***N* (NC)**				**Heterogeneity**		**Egger test^a^**
		**Control means (SD)**	**WMD (95% CI)**	***P*** **-value**	***P*** **-value**	*I*^2^ **(%)**	***P*** **-value**
Milk production, kg/d	11 (24)	21.29 (6.87)	1.348 (0.517; 2.179)	0.001	<0.001	75.86	0.193
**Milk composition, g/100 g**							
Protein	11 (24)	3.71 (0.67)	0.080 (0.045; 0.116)	<0.001	0.004	53.81	0.347
Fat	11 (24)	3.65 (0.88)	0.142 (0.073; 0.211)	<0.001	<0.001	69.98	0.060
Lactose	10 (18)	5.10 (0.95)	0.016 (−0.033; 0.066)	0.517	0.118	26.76	0.132
SCC, × 10^3^ cell/mL	6 (12)	2.52 (0.72)	−0.251 (−0.364; −0.138)	<0.001	<0.001	74.29	0.344

*N*, number of studies; NC, number of comparisons between flavonoids treatment and control treatment; SD, standard deviation; WMD, weighted mean differences between control and treatments with flavonoids; CI, confidence interval of WMD; *p*-value to χ2 (*Q*) test of heterogeneity; *I*^2^, proportion of total variation of size effect estimates that is due to heterogeneity.

a Egger's regression asymmetry test.

SCC, somatic cell count.

### 3.8. Meta-regression and publication bias

Tables 1–6 show no publication bias since the Egger regression asymmetry test was not significant (*p* > 0.05) for any of the variables evaluated. On the other hand, Tables 1–6 show that there was significant (*p* ≤ 0.10) heterogeneity (Q) for DMI, ADG, FCR, HCY, BFT, SOD, CAT, GPx, MDA in blood serum, IgA, IgG, IgM, rumen pH, NH3-N, acetate, propionate, total protozoa, meat pH, CL, ShF, L^*^, protein content, meat IMF and moisture, milk yield, and protein, fat, and SCC content in milk. However, to obtain reliable results, meta-regression analyses are only recommended when the variable of interest is reported in 10 or more studies (46). Consequently, meta-regression analyses were only performed for the following variables: DMI, ADG, rumen pH, acetate, propionate, SOD, milk yield, and milk protein and fat content.

Table 7 shows that the FLAs dose explained (*p* < 0.05) 23.80% of the observed heterogeneity for milk production. The supplementation period explained (*p* < 0.05) 5.90, 28.28, 6.87, and 39.61% of the heterogeneity observed for DMI, ADG, rumen propionate concentration, and milk protein content, respectively. On the other hand, the level of concentrate in the diet explained (*p* < 0.05) 40.10, 37.92, and 10.18% of the heterogeneity observed for ADG, ruminal pH, and milk protein content, respectively. The type of FLAs used explained (*p* < 0.05) between 20.69 and 56.70% of the observed heterogeneity for DMI, rumen pH, SOD, milk yield, milk protein, and milk fat content. Likewise, the FLAs inclusion method explained (*p* < 0.05) 15.12 and 40.45% of the observed heterogeneity for SOD and milk production, respectively. Bovine type explained (*p* < 0.001) 40.60% of the observed heterogeneity for SOD, and days in milk explained (*p* < 0.001) 41.75% of the observed heterogeneity for milk fat content. There was no significant relationship (*p* > 0.05) between the covariates used and the ruminal acetate concentration.

**Table 7:** Meta-regression comparing the associations between covariates and measured outcomes.

**Parameter**	**Covariates**	**QM**	**Df**	***P*-value**	***R*^2^ (%)**
Dry matter intake (DMI)	Flavonoids dose	1.62	1	0.202	0.00
	Supplementation period	12.97	1	<0.001	5.90
	Concentrate level	0.22	1	0.634	0.00
	Flavonoid type	25.68	5	<0.001	29.55
	Method of inclusión	0.85	1	0.654	0.00
	Beef cattle/dairy cattle	0.38	1	0.536	0.00
Average daily gain (ADG)	Flavonoids dose	0.28	1	0.596	0.00
	Supplementation period	5.62	1	0.018	28.28
	Concentrate level	3.89	1	0.049	40.10
	Flavonoid type	6.81	4	0.146	33.47
	Method of inclusion	0.93	1	0.628	0.00
Ruminal pH	Flavonoids dose	1.38	1	0.240	9.18
	Supplementation period	0.08	1	0.772	0.00
	Concentrate level	4.50	1	0.034	37.92
	Flavonoid type	8.49	3	0.037	20.69
	Method of inclusion	0.02	1	0.883	0.00
	Beef cattle/dairy cattle	0.258	1	0.611	0.00
Acetate	Flavonoids dose	0.25	1	0.615	0.00
	Supplementation period	6.98	1	0.108	0.00
	Flavonoid type	2.71	3	0.100	0.00
	Concentrate level	13.45	3	0.104	0.00
	Method of inclusion	1.73	1	0.188	0.00
	Beef cattle/dairy cattle	1.78	1	0.182	0.00
Propionate	Flavonoids dose	1.18	1	0.277	0.00
	Supplementation period	6.77	1	0.009	6.87
	Concentrate level	0.58	1	0.445	0.00
	Flavonoid type	0.99	3	0.803	0.00
	Method of inclusion	1.69	1	0.193	0.00
	Beef cattle/dairy cattle	1.59	1	0.207	0.00
Superoxide dismutase (SOD)	Flavonoids dose	2.11	1	0.146	0.00
	Supplementation period	0.01	1	0.977	3.26
	Concentrate level	0.06	1	0.940	2.55
	Flavonoid type	25.18	4	<0.001	41.84
	Method of inclusion	8.39	1	0.004	15.12
	Beef cattle/dairy cattle	19.66	1	<0.001	40.60
Milk production	Flavonoids dose	4.72	1	0.030	23.80
	Supplementation period	1.40	1	0.236	0.00
	Concentrate level	0.49	1	0.484	0.00
	Flavonoid type	80.93	3	<0.001	35.49
	Method of inclusion	19.19	1	<0.001	40.45
	Days in milk	1.26	1	0.261	0.00
Milk protein	Flavonoids dose	3.21	1	0.073	06.87
	Supplementation period	11.44	1	<0.001	39.61
	Concentrate level	10.84	1	<0.001	10.18
	Flavonoid type	14.03	3	0.003	48.21
	Method of inclusion	1.44	1	0.229	12.92
	Days in milk	0.56	1	0.455	0.00
Milk fat	Flavonoids dose	1.28	1	0.257	0.00
	Supplementation period	0.21	1	0.648	11.24
	Concentrate level	2.97	1	0.085	0.00
	Flavonoid type	18.54	3	<0.001	56.70
	Method of inclusion	0.53	1	0.464	0.00
	Days in milk	17.75	1	<0.001	41.75

QM, coefficient of moderators; QM is considered significant at *p* ≤ 0.05; *R*^2^, the amount of heterogeneity accounted for; df, degree of freedom.

### 3.9. Subgroup analysis

**Figure 2:**
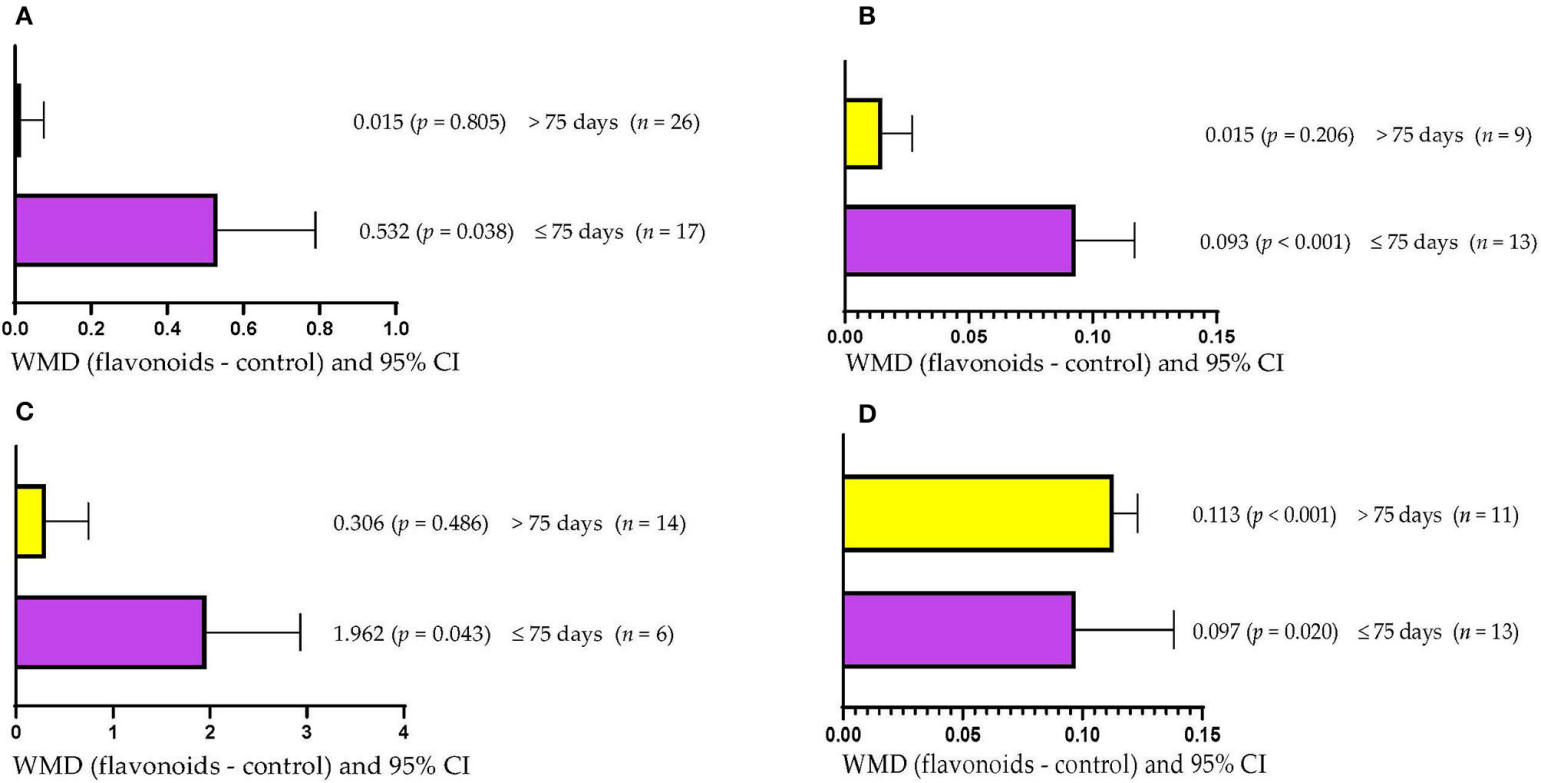
Subgroup analysis [subgroup = supplementation period (days)] of the effect of flavonoids on the diet of the cattle; WMDs, weighted mean differences between flavonoid treatments and control. **(A)** Dry matter intake (DMI), kg/d. **(B)** Average daily gain (ADG), kg/d. **(C)** Propionate, mol/100 mol. **(D)** Milk protein, g/100 g.

DMI increased (WMD = 0.532 kg/d; *p* = 0.038) when dietary supplementation with FLAs lasted up to 75 days (Figure 2A). However, supplementation with FLAs for more than 75 days did not affect DMI (WMD = 0.015 kg/d; *p* = 0.805). Higher ADG (WMD = 0.093 kg/d; *p* < 0.001) was observed when cattle were supplemented with FLAs for periods up to 75 days (Figure 2B). However, when supplementation with FLAs lasted more than 75 days ADG was not affected (WMD = 0.017 kg/d; *p* = 0.206). Ruminal propionate concentration was increased (WMD = 1.962 mol/100 mol; *p* = 0.043) in animals supplemented with FLAs for up to 75 days (Figure 2C). However, ruminal propionate concentration was not affected when FLAs were offered for more than 75 days (WMD = 0.306 mol/100 mol; *p* = 0.486). The protein content in milk increased (*p* < 0.05) regardless of the period of supplementation with FLAs used (Figure 2D). However, the effect was greater when FLAs supplementation lasted longer than 75 days (WMD = 0.113/100 g) than when it lasted up to 75 days (WMD = 0.097/100 g).

**Figure 3:**
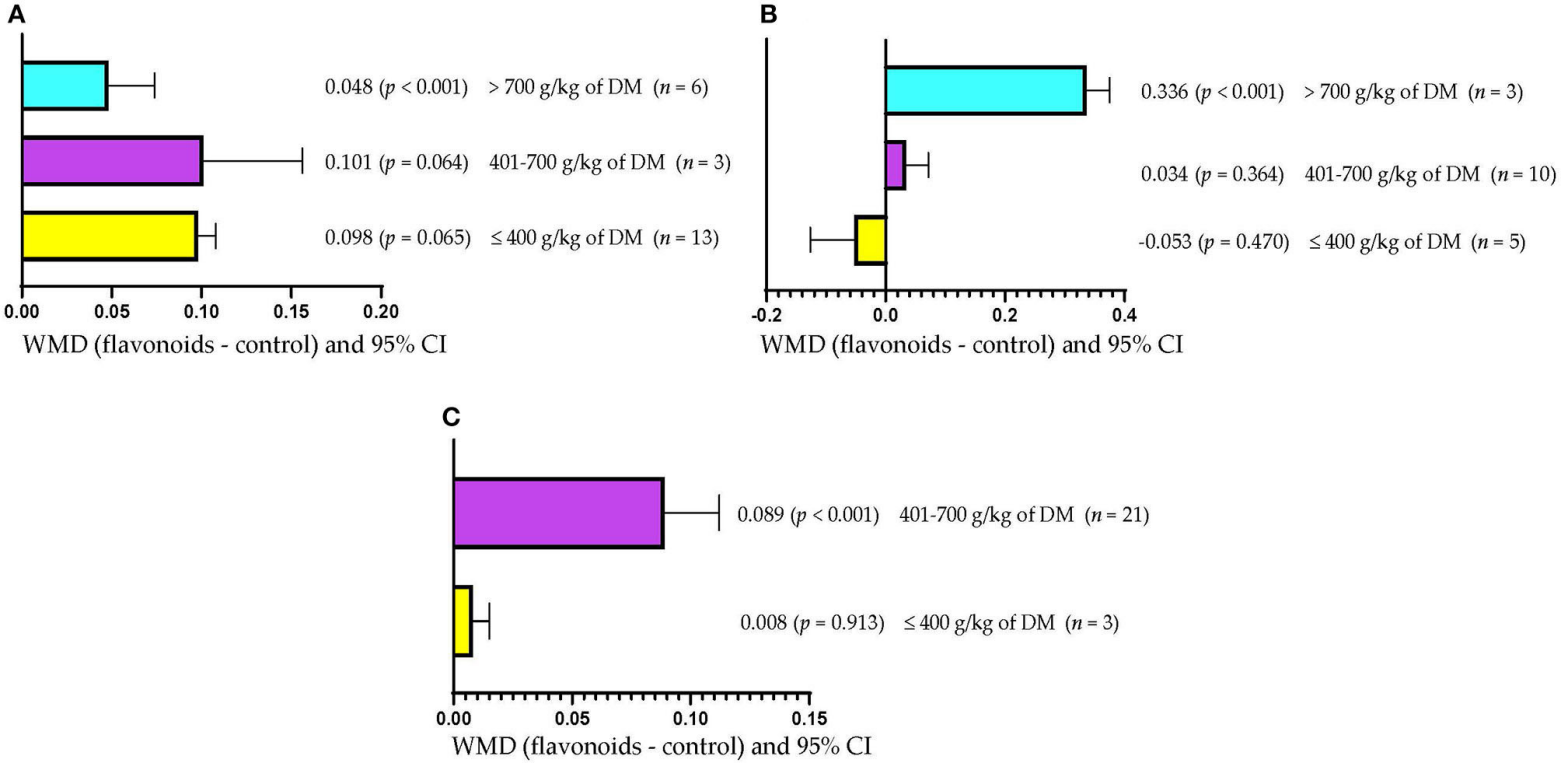
Subgroup analysis [subgroup = concentrate in diet (g/kg of DM)] of the effect of flavonoids on the diet of the cattle; WMDs, weighted mean differences between flavonoid treatments and control. **(A)** Average daily gain (ADG), kg/d. **(B)** Ruminal pH. **(C)** Milk protein, g/100 g.

Figure 3A shows that ADG increased (WMD = 0.048 kg/d; *p* < 0.001) only when FLAs were included in high-concentrate diets (>700 g/kg DM). However, the inclusion of FLAs in diets with low ( ≤ 400 g/kg DM) or moderate (401–700 g/kg DM) concentrate levels did not affect ADG (*p* > 0.05). Rumen pH increased (WMD = 0.336; *p* < 0.001) when FLAs were supplemented in diets with more than 700 g/kg DM of concentrate (Figure 3B). However, the inclusion of FLAs in diets with low (≤400 g/kg DM) or moderate (401–700 g/kg DM) concentrate levels did not affect rumen pH. Figure 3C shows that the inclusion of FLAs in diets with 401–700 g/kg DM of concentrate increased the protein content in milk (WMD = 0.089/100 g; *p* < 0.001). However, milk protein content was not affected when FLAs were fed in low-concentrate diets (≤400 g/kg DM).

**Figure 4:**
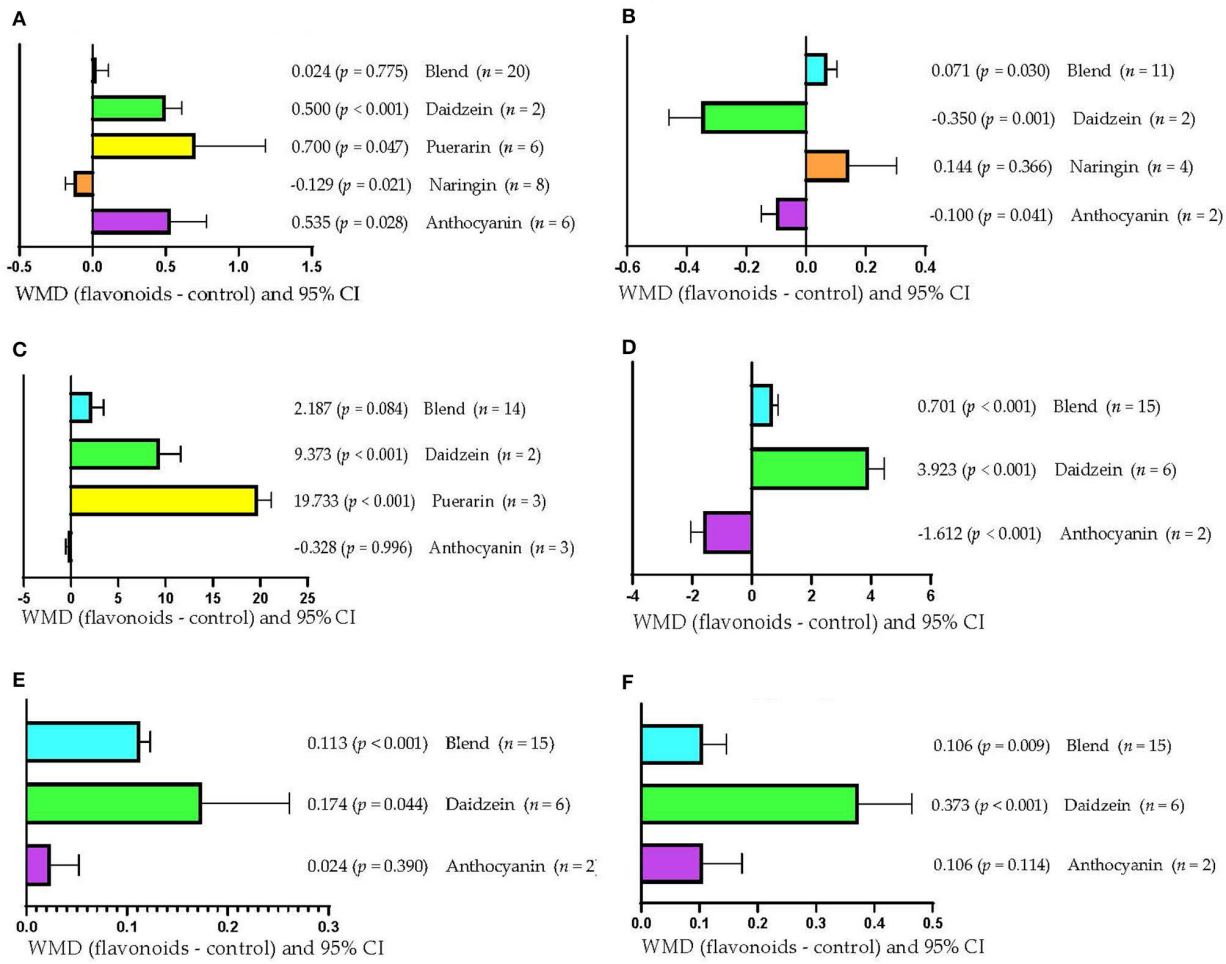
Subgroup analysis (subgroup = flavonoid type) of the effect of flavonoids on the diet of the cattle; WMDs, weighted mean differences between flavonoid treatments and control. **(A)** Dry matter intake (DMI), kg/d. **(B)** Ruminal pH. **(C)** Superoxide dismutase (SOD), U/mL. **(D)** Milk production, kg/d. **(E)** Milk protein, g/100 g. **(F)** Milk fat, g/100 g.

Figure 4A shows that DMI increased (*p* < 0.05) when the type of FLAs used was daidzein (WMD = 0.500 kg/d), puerarin (WMD = 0.700 kg/d), and anthocyanin (WMD = 0.535 kg/d). However, DMI decreased when the type of FLAs used was naringin (WMD = −0.129 kg/d; *p* = 0.021) and was not affected when mixtures of FLAs were used (*p* > 0.05). Rumen pH increased (WMD = 0.071; *p* = 0.030) when FLAs mixtures were used (Figure 4B); however, it decreased when the FLAs used were daidzein (WMD = −0.350; *p* = 0.001) and anthocyanin (WMD = −0.100; *p* = 0.041). Likewise, when the type of FLAs used was naringin, the rumen pH was not affected (*p* > 0.05). Figure 4C shows that the serum concentration of SOD increased (*p* < 0.001) only when the FLAs used were daidzein (WMD = 9.373 U/mL) and puerarin (WMD = 19.733 U/mL). However, the serum SOD concentration was not affected when anthocyanin or FLA mixtures were used (*p* > 0.05). On the other hand, milk production increased (*p* < 0.001; Figure 4D) when mixtures of FLAs (WMD = 0.701 kg/d) and daidzein (WMD = 3.923 kg/d) were used; however, it decreased when the FLAs used were anthocyanins (WMD = −1.612 kg/d; *p* < 0.001). Figure 4E shows that milk protein content increased when mixtures of FLAs (WMD = 0.113/100 g; *p* < 0.001) and daidzein (WMD = 0.174/100 g; *p* = 0.044) were used. However, the protein content in milk was not affected when the FLAs used were anthocyanins (*p* > 0.05). Milk fat content increased (*p* < 0.01; Figure 4F) in response to supplementation with mixtures of FLAs (WMD = 0.106/100 g) and daidzein (WMD = 0.373/100 g); however, it was not affected by anthocyanin supplementation (*p* > 0.05).

**Figure 5:**
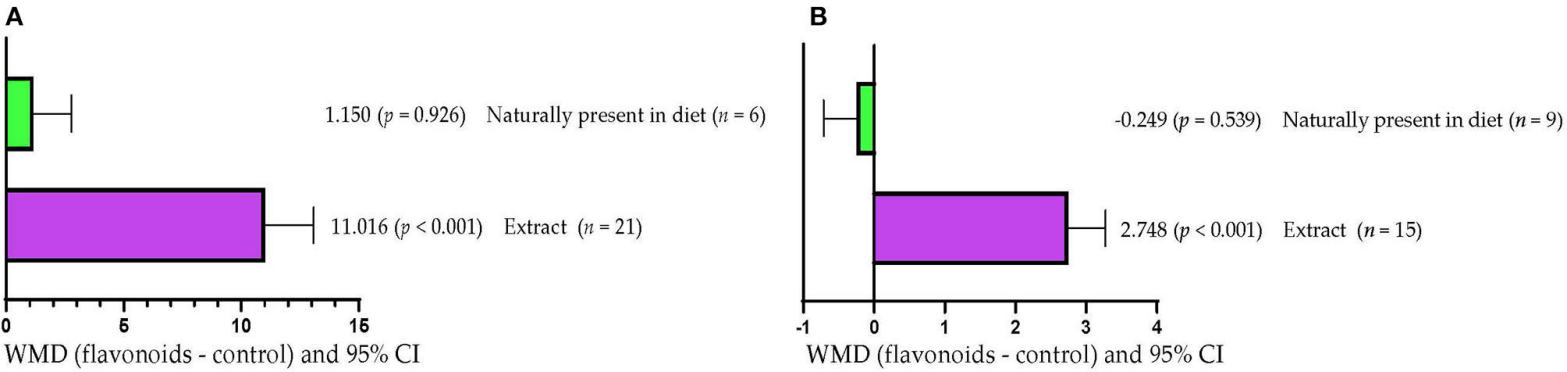
Subgroup analysis [subgroup = method of FLA's inclusion (extract or naturally present in the diet)] of the effect of flavonoids on the diet of the cattle; WMDs, weighted mean differences between flavonoid treatments and control. **(A)** Superoxide dismutase (SOD), U/mL. **(B)** Milk production, kg/d.

Serum SOD concentration increased (WMD =11.016 U/mL; *p* < 0.001) when FLAs extracts were added to diets (Figure 5A). However, when FLAs were supplied as part of the diet ingredients, serum SOD concentration was not affected (*p* > 0.05). Milk production increased (WMD = 2.748 kg/d; *p* < 0.001) in response to supplementation with FLAs extracts (Figure 5B). However, milk production was not affected (*p* > 0.05) when FLAs were supplied as part of the diet ingredients.

**Figure 6:**
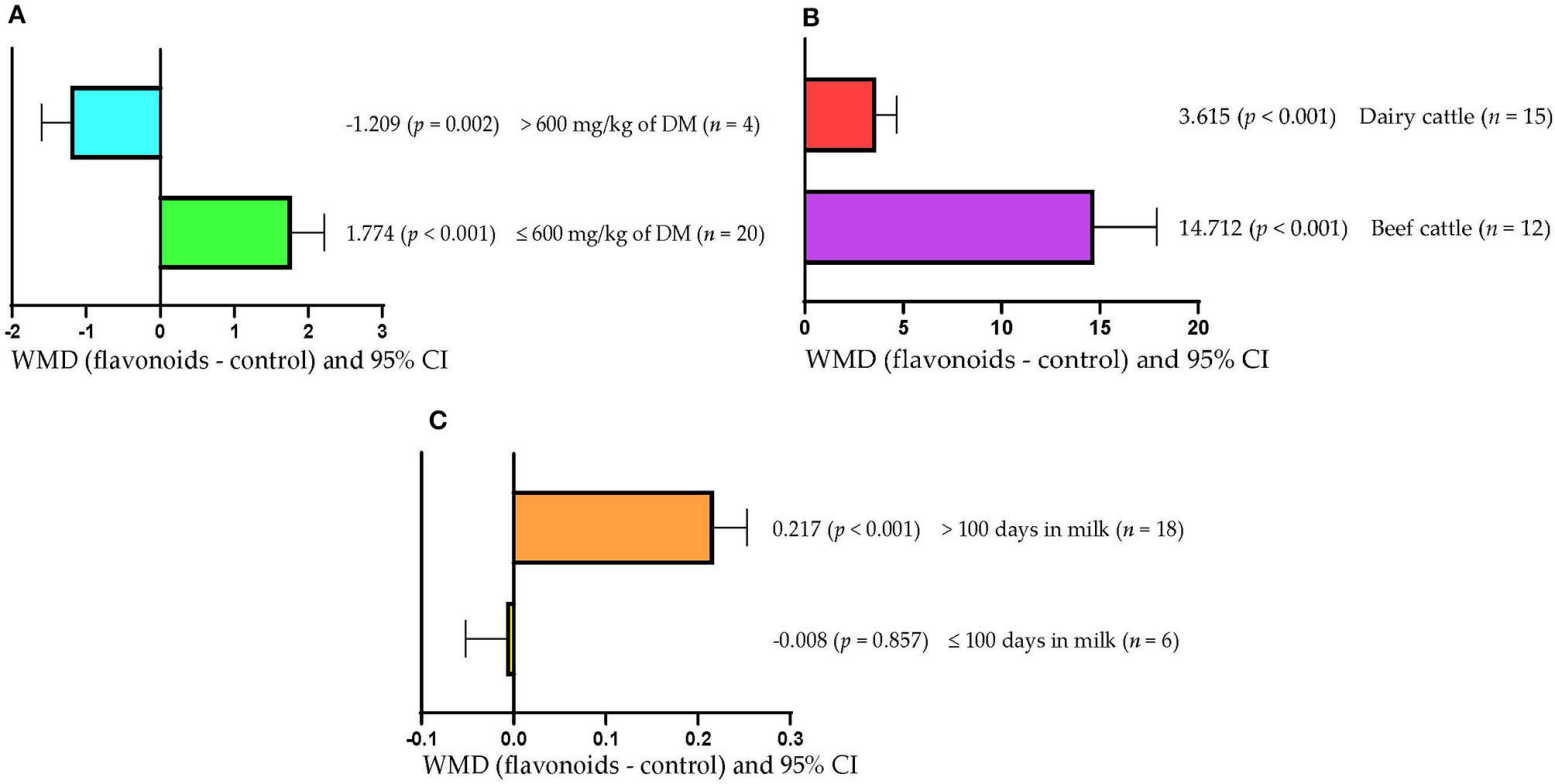
Subgroup analysis [subgroup = flavonoid dose (mg/kg of DM), type of cattle (beef cattle or dairy cattle), and days in milk] of the effect of flavonoids on the diet of the cattle; WMDs, weighted mean differences between flavonoid treatments and control. **(A)** Milk production, kg/d. **(B)** Superoxide dismutase (SOD), U/mL. **(C)** Mil fat, g/100 g.

Figure 6A shows that milk production increased when FLAs doses ≤600 mg/kg DM were used (WMD = 1.774 kg/d; *p* < 0.001). However, milk production decreased (WMD = −1.209 kg/d; *p* = 0.002) when the FLAs doses used were >600 mg/kg DM. In addition, serum SOD concentration increased regardless of the type of bovine used (*p* < 0.001; Figure 6B). However, the effect was greater when FLAs were offered to beef cattle (WMD = 14.712 U/mL) than dairy cows (WMD = 3.615 U/mL). Likewise, milk fat content increased (WMD = 0.217/100 g; *p* < 0.001) when FLAs were offered to cattle that were longer than 100 days in milk (Figure 6C). However, in cattle that were up to 100 days in milk, FLA supplementation did not affect milk fat content (*p* > 0.05).

## 4. Discussion

### 4.1. Dry matter intake and nutrient digestibility

It has been reported that the dietary inclusion of FLAs increases the relative abundance of ruminal bacteria involved in fiber degradation in adult sheep and cattle (20, 47). This effect could increase the rate of passage of feed particles in the rumen and result in higher DMI. In addition, in ruminants (yaks and sheep) dietary supplementation with FLAs increases the relative rumen abundance of the bacterial family *Rikenelleceae* (48, 49), which have a positive correlation with DMI in beef cattle (50). Therefore, similar effects of FLAs supplementation in the present meta-analysis partially explain the observed increase in DMI. On the other hand, in beef cattle, it has been reported that FLAs supplementation reduces the gene expression of bitter taste receptors (TAS2R, such as TAS2R7, TAS2R16, TAS2R38, and TAS2R39) in the rumen (18) and duodenal epithelium (51). This effect could decrease the release of anorexigenic molecules and increase DMI, since the activation of TAS2R triggers the release of anorexigenic molecules, such as cholecystokinin and peptide YY (52, 53). However, a subgroup analysis revealed that naringin supplementation decreased DMI. Naringin is part of the flavanones (a particular class of FLAs), which are abundant in citrus and impart a bitter taste (33). This effect could reduce the food's palatability and explain the lower DMI observed in response to naringin supplementation.

Previous studies (20, 47) have reported that, in adult ruminants (sheep and cattle), dietary supplementation with FLAs increases the relative abundance of ruminal bacteria of the genus *Ruminococcus*. Within this genus are the species *Ruminococcus albus* and *R. flavefaciens*, which play an important role in fiber degradation in the rumen (54). Kim et al. (55) observed that, under *in vitro* conditions, FLAs (catechins) increase the relative abundance of *Fibrobacter succinogenes* bacteria, which are also involved in fiber degradation in the rumen. Low doses (60 mg/kg body weight) of FLAs (mixtures of various types not reported) have been documented to increase the relative abundance of fungi in the rumen of dairy cows by up to 79% (56). According to Akin and Borneman (57), rumen fungi can completely penetrate the cell wall and produce large amounts of cellulases, hemicelluloses, and xylanases, which can increase cellulose degradation. In beef cattle, Niu et al. (58) observed that the dietary inclusion of plants with FLAs increased the relative abundance of rumen bacteria of the genus *Succinivibrio*, which have been positively correlated with NDFD, ADFD, and DMD in cattle (58). Furthermore, Zhao et al. (47) reported that, in growing lambs, supplementation with FLAs (anthocyanins) extracts decreases the relative abundance of ruminal microorganisms of the genus *Prevotella*, which have been negatively correlated with CPD in dairy cows (59). Thus, similar effects of FLAs supplementation in the present study partially explain the observed increases in CPD, NDFD, ADFD, DMD, and OMD.

### 4.2. Growth performance and carcass traits

In the present study, supplementation with FLAs increased DMI, CPD, NDFD, ADFD, EED, OMD, and DMD, which partially explains the higher ADG and lower FCR observed. In dairy cows, Zhan et al. (56) reported that dietary supplementation with FLAs increases the relative abundance of *Tenericutes* and *Mollicutes* rumen microorganisms, which have been positively correlated with ADG in finishing lambs (48). In growing lambs, FLAs supplementation reduces the relative ruminal abundance of the bacterial family *Veillonellaceae* (47), which has a negative correlation with ADG in sheep (60). Du et al. (48) reported that the dietary inclusion of plants containing FLAs increases the relative abundance of the *Rikenellaceae* microbial family in rumen fluid, which has a positive and negative correlation with ADG and FCR in beef cattle, respectively (50). Dorantes-Iturbide et al. (61) reported that, in finishing lambs, supplementation with low doses (1 g/kg DM) of polyherbal additives with FLAs increases up to 23% the efficiency of utilization of dietary energy for weight gain. Furthermore, supplementation with FLAs-rich plants increases muscle protein synthesis in lambs (62). Thus, similar effects of FLAs supplementation in the present study partially explain the observed increase and decrease for ADG and FCR, respectively.

In beef cattle, supplementation with FLAs (200 and 400 mg/kg DM) increases serum levels of IGF-1 (insulin-like growth factor 1) (24), which have a positive correlation (r within 0.61 and 0.67) with ADG in ruminants (63). In addition, in the present meta-analysis, higher serum concentrations of antioxidant enzymes (SOD, CAT, and GPx) and immunoglobulins (IgA, IgG, and IgM) were observed in response to FLAs supplementation. These effects could reduce oxidative stress and improve the health status of the animals, which could result in improved animal performance. On the other hand, a subgroup analysis revealed that ADG was significantly increased when FLAs were offered with high-concentrate diets (>700 g/kg DM). In beef cattle fed high-concentrate diets, FLAs supplementation increases the duodenal flux of microbial protein (64), which may increase metabolic amino acid availability and lead to higher ADG. In addition, previous studies (51, 65) have shown that the dietary inclusion of FLAs (400 mg/kg DM) improves the health of the rumen epithelium in beef cattle fed diets high in concentrate. This effect could result in increased absorption of volatile fatty acids and lead to increased ADG since the rumen epithelium contains papillae that serve as absorptive structures (66).

It has been documented that FLAs supplementation increases the number and diameter of muscle fibers in the ruminant *Longissimus dorsi* muscle (62, 67), which may result in increased LDMA. However, in the present study, FLAs supplementation did not affect LDMA. On the other hand, Liang et al. (25) reported that, in beef cattle, supplementation with FLAs (500 mg/kg DM) increases serum leptin concentration, which has been positively correlated with BFT in beef cattle (68). Consequently, similar effects of FLAs supplementation in the present study partially explain the higher BFT observed. In addition, FLAs promote adipogenesis in the subcutaneous adipose tissue of beef cattle through changes in the expression of several genes (delta like non-canonical notch ligand, insulin like growth factor binding protein 2, wnt family member 6, enhancer binding protein beta, DNA-binding protein inhibitor ID-3, sonic hedgehog protein, and family zinc finger 1) involved in adipogenesis differentiation of subcutaneous adipocytes (69).

### 4.3. Antioxidant status and immune response

According to Celi (5), the excessive accumulation of reactive oxygen species (ROS) causes oxidative stress in ruminants. Shi et al. (70) mentioned that FLAs can be used as natural antioxidants for cattle since they stimulate antioxidant enzymes and eliminate ROS. In the present study, supplementation with FLAs increased the serum levels of SOD, CAT, and GPx. These results suggest that FLAs reduce the oxidative stress caused by ROS in bovines since SOD, CAT, and GPx play an important role in converting ROS into other compounds that are less damaging to the tissues and cells of organisms (10). Furthermore, FLAs have been reported to induce activation of the transcription factor Nrf2 (71), which activates several antioxidant enzymes (10). Consequently, similar effects of FLAs consumption in the present meta-analysis partially explain the observed increases in SOD, CAT, and GPx.

In the present meta-analysis, FLAs supplementation increased TAC in beef and dairy cattle blood serum. This result suggests that the consumption of FLAs improves the total antioxidant status of bovines since TAC considers the total antioxidants present in the blood serum (5). Furthermore, Ghiselli et al. (72) mentions that serum TAC levels obtained after consuming products with antioxidants serve as indicators of the absorption and bioavailability of ingested antioxidants. Consequently, the higher TAC observed in the present study suggests that FLAs consumed by bovines may be absorbed and transferred to the bloodstream to act as blood antioxidants. Furthermore, it has been documented that TAC and ROS serum levels are negatively correlated (73). Therefore, the observed reduction of TAC in the present study suggests that FLAs supplementation decreases ROS in bovine blood serum. On the other hand, supplementation with FLAs decreased the serum concentration of MDA. This result suggests that the consumption of FLAs decreases lipid peroxidation in cattle blood because when lipid peroxidation is low, serum levels of MDA decrease (74).

According to Zhan et al. (75), immunoglobulins are a type of protein with chemical structures similar to antibodies, which participate in the regulation of immune responses. Therefore, obtaining information related to serum immunoglobulin concentrations in ruminants is important since it is an indicator of immunity against pathogenic microorganisms (76). Wolf et al. (77) mention that IgA inhibits the release of inflammatory cytokines, phagocytosis, and antibody-dependent cellular cytotoxicity. In addition, IgM and IgG act against infection since they participate in the phagocytic system and activate the complement system (24). In the present meta-analysis, FLAs supplementation increased serum IgA, IgG, and IgM concentrations, suggesting that FLAs improve immune competence in cattle. The mechanism of action of FLAs on serum immunoglobulin concentrations has not been studied in ruminants. However, FLAs have been documented to increase the expression of genes encoding IgA in mice (78). Likewise, various FLAs increase the number and activity of B1 and B2 lymphocytes (79), which secrete IgG and IgM (80, 81). Therefore, similar effects of FLAs consumption in the present meta-analysis would explain the observed increases in IgA, IgG, and IgM.

### 4.4. Ruminal fermentation

In the present meta-analysis, FLAs supplementation did not affect rumen pH. This result suggests that FLAs do not affect the stability of rumen functions in bovines since rumen pH is an important indicator of internal rumen homeostasis (36, 82). However, a subgroup analysis revealed that rumen pH increased when FLAs were offered in high-concentrate diets (>700 g/kg DM). Under *in vitro* conditions, FLAs decrease the concentration of lactate-producing bacteria (*Streptococcus bovis*) (83). In addition, in beef cattle fed high-concentrate diets, FLAs supplementation increases the abundance of lactate-consuming bacteria (*Megasphera elsdenii* and *Selenomonas rumiantium*) (64, 84). Similar effects of FLAs consumption in the present study could result in a lower rumen lactate concentration, which partially explains the increased rumen pH. On the other hand, the ruminal concentration of NH_3_-N is the primary nitrogenous substrate used by rumen bacteria for microbial protein synthesis (85). Therefore, the absence of changes observed in the present study for the ruminal concentration of NH_3_-N suggests that, in cattle, FLAs supplementation does not affect the synthesis of microbial protein in the rumen. Likewise, the absence of changes observed for NH_3_-N suggests that FLAs supplementation does not affect the balance between rumen ammonia release and uptake.

Balcells et al. (64) mentioned that FLAs supplementation improves rumen fermentation in cattle. In the present study, supplementation with FLAs increased the rumen concentration of propionate with no effect on the concentration of acetate and butyrate. It has been reported that FLAs supplementation decreases the relative abundance of the microbial families *Succiniclasticum* and *Christensenellaceae* (47), which negatively correlates with the rumen concentration of propionate in sheep (67). In addition, under *in vitro* conditions, FLAs increase the relative abundance of the microbial family *Succinivibrionaceae* (86), which positively correlates with the rumen concentration of propionate in beef cattle (87). Therefore, similar effects of FLAs consumption in the present meta-analysis partially explain the increased ruminal propionate concentration. Furthermore, the observed increase in propionate suggests that FLAs increase energy availability for growth and production in cattle, since ruminal propionate is the main precursor of gluconeogenesis in ruminants (88).

FLAs supplementation decreased the ruminal concentration of total protozoa. This effect could improve the utilization efficiency of the protein and energy consumed by bovines since the reduction of rumen protozoa leads to less rumen protein degradation (89) and decreases enteric methane emissions (90).

### 4.5. Meat quality

The supplementation with FLAs did not affect the meat's pH or CL. These results indicate that the FLAs do not affect the quality or the water-holding capacity (WHC) of beef since the pH and CL serve as indicators to evaluate the quality (91) and WHC of the meat (92), respectively. On the other hand, lower ShF and MDA were observed in beef cattle meat in response to FLAs supplementation. These results indicate that FLAs improve beef's tenderness and oxidative stability, as ShF and MDA are indicators of meat tenderness (93) and lipid peroxidation (94), respectively. The lower ShF could be related to the reduction in IMF observed in beef from bovines supplemented with FLAs, since there is a negative correlation (*r* = −0.54) between ShF and IMF in beef (95). In beef cattle, low doses (400 mg/kg DM) of FLAs have been reported to decrease skeletal muscle fiber diameter (67), which is positively correlated with ShF in beef (96). The reduction observed in the present study for lipid peroxidation of meat partially explains the lower ShF, as oxidation decreases post-mortem calpain activity and myofibrillar proteolysis, leading to higher ShF (97).

The reduction observed for MDA in meat indicates that FLAs supplementation improves beef's quality and shelf life because when oxidation reactions in meat increase, the quality, and shelf-life decrease (98). Previous studies (62, 99) have reported that FLAs supplementation increases the activity of SOD, CAT, and GPx in the *Longissimus dorsi* muscle of small ruminants. Therefore, similar effects of FLAs consumption in the present meta-analysis partially explain the lower MDA content observed. On the other hand, it is widely documented that meat color is a crucial factor that consumers consider when choosing fresh meat (93). L^*^ and a^*^ values are related to meat brightness and metmyoglobin content, respectively (93, 100). In the present meta-analysis, supplementation with FLAs did not affect L^*^ and a^*^ in meat, indicating that FLAs do not affect metmyoglobin formation or appearance in beef. Furthermore, the lower b^*^ observed in response to FLAs supplementation is positive, as consumers expect to find low b^*^ values in fresh meat (101).

FLAs supplementation did not affect the meat's protein, moisture, and ash content; however, the IMF increased. These results indicate that FLAs do not negatively affect the nutritional value of beef since the protein and ash content of the meat are related to its nutritional value (93, 102). In contrast, the higher IMF observed could be positive since IMF correlates positively with beef's tenderness and juiciness (103). In addition, some FLAs increase the adipogenesis of bovine preadipocytes (104), which participate in the deposition of IMF (105). In pigs, FLAs supplementation increases skeletal muscle PPARγ mRNA expression levels (106), which positively correlates with IMF (107). Similar effects of FLAs consumption in the present study partially explain the higher IMF observed.

### 4.6. Milk production and quality

It has been mentioned that increasing the utilization efficiency of ingested feed is necessary to improve milk production in ruminants (108). In the present study, FLAs supplementation increased DMD, OMD, CPD, NDFD, ADFD, and EED. These results indicate that FLAs increase the utilization efficiency of ingested feed and partially explain the higher milk production observed in response to FLAs supplementation. In addition, the higher milk production could be related to the increased ruminal propionate concentration observed since milk production in dairy cows increases curvilinearly in response to the supply of gluconeogenic precursors (109). In lactating buffaloes, it has been reported that supplementation with FLAs-rich plants increases serum somatotropin levels by up to 50% (110), which positively correlates with milk production in dairy cows (111). It has been documented that FLAs decrease the ruminal abundance of *Clostridium* microorganisms (112), which negatively correlates with milk production in bovines (113). In growing sheep, FLAs supplementation increases the relative abundance of the *Ruminococcaceae* microbial family (47), which positively correlates with milk production in dairy cows (113). Consequently, similar effects of FLAs consumption in the present meta-analysis partially explain the higher milk production observed.

Higher protein and fat content in milk was observed in response to FLAs supplementation. Under *in vitro* conditions, FLAs decrease the relative abundance of *Clostridium* (112) and *Methanobrevibacter* spp. (114), which negatively correlates with the percentage of milk protein in ruminants (115, 116). In beef cattle, FLAs supplementation increases the ruminal presence of the microbial family *Succinivibrionaceae* (57), which positively correlates with the protein content in milk from dairy cows (116). In the present study, the FLAs decreased the ruminal concentration of total protozoa, which negatively correlates with the fat content in the milk of small ruminants (117). In dairy cows, Kong et al. (59) detected that FLAs supplementation increases the relative rumen abundance of the microbial genus *Butyrivibrio*, which has a positive correlation with the fat percentage in dairy cows (116). In dairy goats, FLAs supplementation increases the expression of genes involved in milk fat synthesis, such as genes related to *de novo* fatty acid synthesis [acetyl-CoA carboxylase α (ACACA), fatty acid synthase (FASN), and stearoyl-CoA desaturase (SCD1)] and triglyceride synthesis [diacylglycerol Oacyltransferase 1 (DGAT1), diacylglycerol O-acyltransferase 2 (DGAT2), and glycerol-3-phosphate acyltransferase 1 (GPAM)] (118). Briefly, acetyl-CoA and malonyl-CoA are condensed under FASN catalysis, two carbon atoms are added to the carboxyl of the fatty acid, the ACACA gene limits the rate of the process, and the SCD1 gene catalyzes the synthesis of monounsaturated fatty acids (118). Likewise, the GPAM gene catalyzes the acyl group transfer from acyl-CoA to generate 1-acylglycerol-3-phosphate, while the GDAT1 and DGAT2 genes catalyze the formation of triglycerides with fatty acyl-CoA (118). Therefore, similar effects of FLAs consumption in the present study partially explain the increased milk fat and protein content observed. On the other hand, FLAs supplementation did not affect the lactose content in milk. This result was not expected since the FLAs increased the rumen concentration of propionate, which is the primary short-chain fatty acid required for lactose biosynthesis (108).

Tong et al. (119) mention that SCC is a widely used indicator to assess the health of the mammary gland and the quality of milk in bovines. For example, an increase in SCC is associated with intramammary infection and negatively affects raw milk quality (120). In the present meta-analysis, lower SCC was observed in response to FLAs supplementation, indicating that FLAs improve mammary gland health and milk quality in cattle. In dairy cows, FLAs supplementation decreases the presence of *Staphylococcus* bacteria in milk (121), which positively correlates with SCC in ruminant milk (122). In addition, IgA has been reported to be involved in the protection of mucous membranes, IgM is the first line of defense against infections, and IgG plays an important role in the immune response against infections (75, 121). In the present meta-analysis, IgG, IgA, and IgM serum levels increased in response to FLAs supplementation. Therefore, similar effects of FLAs consumption in the present study partially explain the lower SCC observed.

### 4.7. Limitations and strengths of the meta-analysis

The present meta-analysis was limited to research conducted only in beef cattle and dairy cows and may not apply to other ruminant species. In addition, high heterogeneity was detected in most of the response variables evaluated, which may represent a limitation in applying the global results obtained. However, this problem was diminished with the use of subgroup analysis, which allowed us to identify the specific conditions under which FLAs could be used successfully to improve different important parameters in beef cattle and dairy cows. Finally, this meta-analysis also establishes the steps for implementing future standardized experimental designs on the use of FLAs as growth promoters and natural antioxidants in beef cattle and dairy cows.

## 5. Conclusions

The results obtained in the present meta-analysis indicate that FLAs can be used as natural growth promoters in beef cattle and, at the same time, improve feed conversion. The best result for daily weight gain is obtained with FLAs supplementation periods up to 75 days and diets high in concentrate (>700 g/kg DM). Likewise, including FLAs in bovine diets improves dry matter intake and nutrient digestibility. The best dry matter intake is obtained with periods up to 75 days and when the FLAs used are puerarin, anthocyanin, and daidzein. Furthermore, supplementation with FLAs improves total antioxidant status and immune response in cattle by reducing serum concentration of malondialdehyde and increasing serum levels of antioxidant enzymes and immunoglobulins. The best results for serum concentration of superoxide dismutase are obtained with FLAs extracts and when the FLAs used are puerarin or daidzein. At the same time, FLAs supplementation improves meat quality by reducing shear force and malondialdehyde content. In addition, FLAs improve milk production and composition. The highest milk production is obtained when FLAs extracts are used, with daidzein or mixtures of FLAs, and low doses of FLAs (≤600 mg/kg DM). The best results for milk protein content are obtained with supplementation periods longer than 75 days, diets with moderate levels of concentrate (400–700 g/kg DM), and daidzein or mixtures of FLAs. Likewise, the best fat content in milk is achieved with daidzein or mixtures of FLAs and using cows with more than 100 days in milk. Finally, FLAs supplementation improves ruminal fermentation in cattle through increased ruminal propionate concentration and reduced total rumen protozoa. The best rumen propionate concentration is obtained with supplementation periods of up to 75 days.

## Data availability statement

The raw data supporting the conclusions of this article will be made available by the authors, without undue reservation.

## Author contributions

JO-O: conceptualization, methodology, data curation, formal analysis, investigation, visualization, writing—original draft preparation, and writing—review and editing. GD-I: methodology, data curation, formal analysis, validation, and writing—review and editing. AL-B: conceptualization, resources, writing—review and editing, supervision, project administration, resources, and funding acquisition. AC-C: methodology, investigation, data curation, and writing—review and editing. LM-R: data curation, supervision, and writing—review and editing. GM-M: software, supervision, and writing—review and editing. All authors contributed to the article and approved the submitted version.
