# Supplementary material for: Meta-analysis of flavonoids use into beef and dairy cattle diet: Performance, antioxidant status, ruminal fermentation, meat quality, and milk composition
Source: Front Vet Sci. 2023 Feb 15;10:1134925. doi: 10.3389/fvets.2023.1134925 (PMC9975267; doi:10.3389/fvets.2023.1134925)
Supplement: Supplementary file 1 [file Data_Sheet_1.pdf]

Supplementary Material

Meta-analysis of flavonoids use into beef and dairy cattle diet: Performance, antioxidant status, ruminal fermentation, meat quality, and milk composition

José Felipe Orzuna-Orzuna [1], Griselda Dorantes-Iturbide [1], Alejandro Lara-Bueno [1,*], Alfonso Juventino Chay-Canul [2], Luis Alberto Miranda-Romero [1] and Germán David Mendoza-Martínez [3]

*** Correspondence:** Alejandro Lara-Bueno: alarab_11@hotmail.com

Supplementary Table S1. Summary of the studies included in the meta-analysis

Author	Country	Bovine type	Flavonoid type	Method of inclusion
Aguiar et al. (27)	Brazil	Dairy cattle	Blend (*n* = 3)	Naturally present (*n* = 3)
Balcells et al. (64)	Spain	Beef cattle	Naringin	Extract
Chen et al. (123)	China	Beef cattle	Puerarin (*n* = 5)	Extract (*n* = 5)
Cherdthong et al. (85)	Thailand	Beef cattle	Blend (*n* = 3)	Naturally present (*n* = 3)
Gao et al. (124)	China	Beef cattle	Anthocyanin	Naturally present
Gao et al. (125)	China	Beef cattle	Anthocyanin	Naturally present
Gessner et al. (126)	Germany	Dairy cattle	Blend	Extract
Hosoda et al. (127)	Japan	Dairy cattle	Anthocyanin	Naturally present
Jiménez-Ocampo et al. (128)	Mexico	Beef cattle	Naringin (*n* = 2)	Extract (*n* = 2)
Jing-Wei et al. (129)	China	Dairy cattle	Blend	Extract
Kang et al. (130)	Korea	Beef cattle	Quercetin (*n* = 2)	Extract (*n* = 2)
Li et al. (67)	China	Beef cattle	Puerarin (*n* = 3)	Extract (*n* = 3)
Liang et al. (20)	China	Beef cattle	Daidzein (*n* = 2)	Extract (*n* = 2)
Liang et al. (25)	China	Beef cattle	Daidzein (*n* = 2)	Extract (*n* = 2)
Liang et al. (69)	China	Beef cattle	Daidzein	Extract
Liu et al. (15)	China	Dairy cattle	Daidzein (*n* = 6)	Extract (*n* = 6)
Ma et al. (131)	China	Dairy cattle	Catechin	Extract
Matsuba et al. (132)	Japan	Dairy cattle	Anthocyanin	Naturally present
Paniagua et al. (65)	Spain	Beef cattle	Naringin (*n* = 2)	Extract (*n* = 2)
Paniagua et al. (18)	Spain	Beef cattle	Naringin	Extract
Paniagua et al. (19)	Spain	Beef cattle	Naringin	Extract
Paniagua et al. (51)	Spain	Beef cattle	Naringin	Extract
Peng et al. (26)	China	Beef cattle	Puerarin (*n* = 3)	Extract (*n* = 3)
Prommachart et al. (133)	Thailand	Beef cattle	Anthocyanin (*n* = 3)	Naturally present (*n* = 3)
Prommachart et al. (134)	Thailand	Beef cattle	Anthocyanin (*n* = 3)	Naturally present (*n* = 3)
Santos et al. (135)	Brazil	Dairy cattle	Blend (*n* = 2)	Naturally present (*n* = 2)
Seradj et al. (84)	Spain	Beef cattle	Blend	Extract
Tilahun et al. (16)	China	Dairy cattle	Blend (*n* = 3)	Naturally present (*n* = 3)
Tilahun et al. (23)	China	Dairy cattle	Blend (*n* = 3)	Naturally present (*n* = 3)
Totakul et al. (2022)	Thailand	Dairy cattle	Blend (*n* = 2)	Naturally present (*n* = 2)
Valero et al. (136)	Brazil	Beef cattle	Blend	Naturally present
Valero et al. (137)	Brazil	Beef cattle	Blend	Naturally present
Valero et al. (138)	Brazil	Beef cattle	Blend	Naturally present
Zhan et al. (75)	China	Dairy cattle	Blend (*n* = 3)	Extract (*n* = 3)
Zhao et al. (139)	China	Beef cattle	Daidzein	Extract

Zhao et al. (24)	China	Beef cattle	Daidzein	Extract

n: number of treatments.

References of the studies included in the meta-analysis and that were not cited and written in the references of the manuscript.

123. Chen H, Peng T, Shang H, Shang X, Zhao X, Qu M, Song X. RNA-Seq analysis reveals the potential molecular mechanisms of Puerarin on intramuscular fat deposition in heat-stressed beef cattle. Front Nutr (2022) 9: 817557. doi: 10.3389/fnut.2022.817557

124. Gao J, Cheng B, Liu Y, Li MM, Zhao G. Dietary supplementation with red cabbage extract rich in anthocyanin increases urinary hippuric acid excretion and consequently decreases nitrous oxide emissions in beef bulls. Anim Feed Sci Technol (2021) 281: 115075. doi: 10.1016/j.anifeedsci.2021.115075

125. Gao J, Cheng BB, Liu YF, Li MM, Zhao GY. Effects of red cabbage extract rich in anthocyanins on rumen fermentation rumen bacterial community nutrient digestion and plasma indices in beef bulls. Animal (2022) 16: 100510. doi: 10.1016/j.animal.2022.100510

126. Gessner DK, Koch C, Romberg FJ, Winkler A, Dusel G, Herzog E, Most E, Eder K. The effect of grape seed and grape marc meal extract on milk performance and the expression of genes of endoplasmic reticulum stress and inflammation in the liver of dairy cows in early lactation. J Dairy Sci (2015) 98: 8856–8868. http://dx.doi.org/10.3168/jds.2015-9478

127. Hosoda K, Eruden B, Matsuyama H, Shioya S. Effect of anthocyanin-rich corn silage on digestibility milk production and plasma enzyme activities in lactating dairy cows. Anim Sci J (2012) 83: 453–459. doi: 10.1111/j.1740-0929.2011.00981.x

128. Jiménez-Ocampo R, Montoya-Flores MD, Herrera-Torres E, Pámanes-Carrasco G, Arceo-Castillo JI, Valencia-Salazar SS, Arango J, Aguilar-Pérez CF, Ramírez-Avilés L, Solorio-Sánchez FJ, Piñeiro-Vázquez ÁT, Ku-Vera JC. Effect of chitosan and naringin on enteric methane emissions in crossbred heifers fed tropical grass. Animals (2021) 11: 1599. doi: 10.3390/ani11061599

129. Jing-Wei Z, Yi-Yuan S, Xin L, Hua Z, Hui N, Luo-Yun F, Ben-Hai X, Jin-Jin T, Lin-Shu J. Microbiome and metabolic changes of milk in response to dietary supplementation with bamboo leaf extract in dairy cows. Front Nutr (2021) 8: 723446. doi: 10.3389/fnut.2021.723446

130. Kang M, Kim HJ, Jang A, Gam DK, Yun GS, Jo C. Effect of dietary supplementation of quercetin on antioxidant activity and meat quality of beef cattle. Korean J Agric Sci (2012) 39: 61–68. doi: 10.7744/cnujas.2012.39.1.061

131. Ma Y, Feng Y, Song L, Li M, Dai H, Bao H, Zhang G, Zhao L, Zhang C, Yi J, Liang Y. Green tea polyphenols supplementation alters immunometabolism and oxidative stress in dairy cows with hyperketonemia. Anim Nutr (2021) 7: 206–215. doi: 10.1016/j.aninu.2020.06.005

132. Matsuba T, Kubozono H, Saegusa A, Obata K, Gotoh K, Miki K, Akiyama T, Oba M. Short communication: Effects of feeding purple corn (Zea mays L.) silage on productivity and blood superoxide dismutase concentration in lactating cows. J Dairy Sci (2019) 102: 7179–7182. doi: 10.3168/jds.2019-16353

133. Prommachart R, Uriyapongson J, Cherdthong A, Uriyapongson S. Feed intake nutrient digestibility antioxidant activity in plasma and growth performance of male dairy cattle fed black rice and purple corn extracted residue. Trop Anim Sci J (2021) 44: 307–315. doi: 10.5398/tasj.2021.44.3.307

134. Prommachart R, Cherdthong A, Navanukraw C, Pongdontri P, Taron W, Uriyapongson J, Uriyapongson S. Effect of dietary anthocyanin-extracted residue on meat oxidation and fatty acid profile of male dairy cattle. Animals (2021) 11: 322. doi: 10.3390/ani11020322

135. Santos GT, Lima LS, Schogor ALB, Romero JV, De Marchi FE, Grande PA, Santos NW, Santos FS, Kazama R. Citrus pulp as a dietary source of antioxidants for lactating holstein cows fed highly polyunsaturated fatty acid diets. Asian-Australas J Anim Sci (2014) 27: 1104-1113. doi: 10.5713/ajas.2013.13836

136. Valero VM, do Prado RM, Zawadzki F, Eiras CM, Madrona GS, do Prado IN. Propolis and essential oils additives in the diets improve animal performance and feed efficiency of bulls finished in feedlot. Acta Sci Anim Sci (2014) 36: 419–426. doi: 10.4025/actascianimsci.v36i4.23856

137. Valero MV, Torrecilhas JA, Zawadzki F, Bonafé EG, Madrona GS, Prado RMD, Passetti RAC, Rivaroli DC, Visentainer JV, Prado IND. Propolis or cashew and castor oils effects on composition of Longissimus muscle of crossbred bulls finished in feedlot. Chil J Agric Res (2014) 74: 445–451. http://dx.doi.org/10.4067/S0718-58392014000400011

138. Valero MV, Farias MS, Zawadzki F, Prado RM, Fugita CA, Rivaroli DC, Ornaghi MG, Prado IN. Feeding propolis or essential oils (cashew and castor) to bulls: Performance digestibility and blood cell counts. Rev Colom Cienc Pecua (2016) 29: 33–42. doi: 10.17533/udea.rccp.v29n1a04

139. Zhao XH, Yang ZQ, Bao LB, Wang CY, Zhou S, Gong JM, Fu CB, Xu LJ, Liu CJ, Qu M. Daidzein enhances intramuscular fat deposition and improves meat quality in finishing steers. Exp Biol Med (2015) 88: 215–227. doi: 10.1177/1535370214564

Supplementary Table S2. Descriptive statistics of the complete data set for the effect of FLAs supplementation on beef and dairy cattle diets.

**Parameter**		**Mean**		**Median**		**Minimum**		**Maximum**		**SD**	
Dietary features	NC	Control	FLAs	Control	FLAs	Control	FLAs	Control	FLAs	Control	FLAs
Concentrate, g/kg DM	75	576.0	576.0	500.0	500.0	150.0	150.0	950.0	950.0	214.0	214.0
FLAs, mg/kg DM	77	-	606.6	-	400.0	-	12	-	3104.0	-	809.0
Days in milk	27	116.0		126.0		7.0		164.0		46.63	
Duration, days	77	75.0		70.0		24.0		168.0		36.5	

NC = number of comparisons; FLAs = flavonoids; SD = standard deviation; DM = dry matter.
